# Rates, risks and routes to reduce vascular dementia (R4vad), a UK-wide multicentre prospective observational cohort study of cognition after stroke: Protocol

**DOI:** 10.1177/2396987320953312

**Published:** 2020-10-11

**Authors:** Joanna M Wardlaw, Fergus Doubal, Rosalind Brown, Ellen Backhouse, Lisa Woodhouse, Philip Bath, Terence J Quinn, Thompson Robinson, Hugh S Markus, Richard McManus, John T O’Brien, David J Werring, Nikola Sprigg, Adrian Parry-Jones, Rhian M Touyz, Steven Williams, Yee-Haur Mah, Hedley Emsley, the R4VaD Investigators

**Affiliations:** 1 Centre for Clinical Brain Sciences, University of Edinburgh, Edinburgh, UK; 2 UK Dementia Research Institute, University of Edinburgh, Edinburgh, UK; 3 Stroke Trials Unit, Division of Clinical Neuroscience, University of Nottingham, Nottingham, UK; 4 Institute of Cardiovascular and Medical Sciences, University of Glasgow, UK; 5 Department of Cardiovascular Sciences and NIHR Leicester Biomedical Research Centre, University of Leicester, UK; 6 Department of Neurology, University of Cambridge, Cambridge, UK; 7 Department of General Practice, University of Oxford, UK; 8 Department of Psychiatry, University of Cambridge, Cambridge, UK; 9 National Hospital for Neurology and Neurosurgery, London, UK; 10 NHS Foundation Trust and Stroke Research Centre, University College Hospitals, London, UK; 11 Institute of Neurology, University College, London, UK; 12 Division of Cardiovascular Sciences, School of Medicine, Faculty of Biology, Medicine & Health, Manchester Academic Health Science Centre, The University of Manchester, Manchester, UK; 13 King’s College Hospital NHS Foundation Trust, School of Biomedical Engineering and Imaging Sciences, King’s College London, UK; 14 Department of Neurology, Lancashire Teaching Hospitals NHS Foundation Trust & Lancaster Medical School, Lancaster University, UK

**Keywords:** Stroke, cognition, dementia, observational

## Abstract

**Background:**

Stroke commonly affects cognition and, by definition, much vascular dementia follows stroke. However, there are fundamental limitations in our understanding of vascular cognitive impairment, restricting understanding of prevalence, trajectories, mechanisms, prevention, treatment and patient-service needs.

**Aims:**

Rates, Risks and Routes to Reduce Vascular Dementia (R4VaD) is an observational cohort study of post-stroke cognition. We aim to recruit a wide range of patients with stroke, presenting to geographically diverse UK hospitals, into a longitudinal study to determine rates of, and risk factors for, cognitive and related impairments after stroke, to assess potential mechanisms and improve prediction models.

**Methods:**

We will recruit at least 2000 patients within six weeks of stroke with or without capacity to consent and collect baseline demographic, clinical, socioeconomic, lifestyle, cognitive, neuropsychiatric and informant data using streamlined patient-centred methods appropriate to the stage after stroke. We will obtain more detailed assessments at four to eight weeks after the baseline assessment and follow-up by phone and post yearly to at least two years. We will assess diagnostic neuroimaging in all and high-sensitivity inflammatory markers, genetics, blood pressure and diffusion tensor imaging in mechanistic sub-studies.

**Planned outputs:** R4VaD will provide reliable data on long-term cognitive function after stroke, stratified by prior cognition, stroke- and patient-related variables and improved risk prediction. It will create a platform enabling sharing of data, imaging and samples. Participants will be consented for re-contact, facilitating future clinical trials and providing a resource for the stroke and dementia research communities.

## Introduction and rationale

Stroke and dementia share many risk factors,^1^ and each is a risk factor for the other.^2,3^ Stroke and transient ischaemic attack (TIA) increase the risk of post-stroke cognitive impairment (PSCI) and vascular dementia (VaD), but risk-prediction for individuals remains difficult.^2,4^ There are limited data on rates and progression of PSCI by time and clinically relevant strata that account for pre-morbid and pre-stroke cognition, medical, lifestyle and socioeconomic factors,^2^ but improved understanding of these predictors would improve risk stratification, identify mechanisms and intervention targets.^1^

The limited progress to date in predicting, understanding and ameliorating PSCI reflects the variability of stroke presentations, its consequences and co-morbidities. Nonetheless, post-stroke cognitive impairment is a common, under-researched problem and a priority for patients, carers, health services, funders, policy makers and governments in recent years.^5^

*Rates, Risks and Routes to Reduce Vascular Dementia* (R4VaD) is a Priority Programme in VaD funded by the Stroke Association, British Heart Foundation and Alzheimer’s Society. It is a large, multicentre, longitudinal, inclusive study in patients presenting with stroke or TIA to UK Stroke Centres, using standardised proportionate ascertainment methods to assess cognition, functional and neuropsychiatric outcomes up to at least two years after stroke.

## Methods and design

### Study aims and objectives

The primary aim of R4VaD is to determine rates of cognitive impairment and dementia up to at least two years after stroke across a wide range of stroke severities and participant demographics including socioeconomic status (SES) and premorbid cognitive status.

Our secondary aims are to:

1. Identify key risk predictors and improve risk prediction model precision for individuals;
2. Improve understanding of mechanisms of PSCI;
3. Improve cognitive testing for PSCI;
4. Provide data to inform the design of future RCTs and help plan clinical services for patients with PSCI;
5. Establish a well-phenotyped population, in follow-up, with consent for re-contact for future studies and trials.

The objectives are to:

1. Recruit a large (>2000) sample of patients post-stroke/TIA and perform baseline and then regular comprehensive follow-up assessments to at least two years post-stroke.
2. Classify cognitive outcomes into subtypes characterised by patient-related factors.
3. Construct and refine a comprehensive, flexible, patient-focussed and patient-sensitive test battery during the study.
4. Identify risk-stratification scores for patients’ post-stroke cognitive function to inform outcome event rates and provide data for sample-size calculations for trials.
5. Synthesise a registry of participants willing to consider taking part in future studies/trials.

### Ethics and regulatory approvals

R4VaD is approved by Ethics Committees in Scotland (A Research Ethics Committee; Ref number 18/SS/055), England (Health Research Authority), Wales (Health and Care Research) and Northern Ireland (all Northeast Newcastle and North Tyneside 1; Ref number 18/NE/0150). NHS Research and Innovation Office approval is given in each participating site. The study is adopted by the National Institute for Health Research (NIHR) Clinical Research Network in England, Wales and Northern Ireland and the Stroke Research Network in Scotland.

## Patients

### Inclusion and exclusion criteria

We aim to recruit at least 2000 participants from UK stroke centres in all four nations. We will include all adult (age 18 or over) patients with ischaemic or intracerebral haemorrhage (ICH) or TIA, who are expected to survive to at least 12 weeks after stroke, and whether or not they have a diagnosis of cognitive impairment or dementia prior to stroke, with or without capacity to consent. We exclude patients with subarachnoid haemorrhage (Table 1).

**Table 1.** Inclusion and exclusion criteria.

Inclusion criteria
• Patients aged ≥18, with no upper age• Ischaemic, or spontaneous haemorrhagic (non-traumatic, non-subarachnoid haemorrhage, non-AVM) stroke and transient ischaemic attack (TIA; where feasible^a^) with no severity limit• Expected to survive at least to 12 weeks
Exclusion criteria
• Inclusion criteria are not met, in particular, at onset, the patient is not expected to survive more than 12 weeks• Aneurysmal, traumatic or AVM-associated haemorrhage or subarachnoid haemorrhage• Stroke mimics such as brain tumours

^a^Patients with TIA may be recruited where the research resource is sufficient, but patients with stroke remain the priority where research resource is restricted.

### Participant identification, recruitment and consent

Participants are recruited from in- and out-patient stroke services. Informants, usually a close family member, are also requested.

Participants with capacity (and informants where available) give informed written consent at study entry prior to any study-related procedures. Patients without capacity have consent, assent, opinion or waiver of consent obtained from the appropriate person within each jurisdiction since guardianship for persons without capacity differs in Scotland, Northern Ireland, England and Wales. The appropriate person may also act as the informant. Patients who regain capacity and wish to remain in the study provide written informed consent. Where participants lose capacity during the study, we continue to collect data but seek consent for this from the appropriate consultee, relative, friend, welfare attorney, as defined by jurisdiction. If participants withdraw, we retain data collected to that point and record reasons for withdrawal if available. Consent includes the ability to undertake trial procedures remotely, i.e. to use phone or postal contact, if needed due to patient circumstance or national policy.

### Co-enrolment

Co-enrolment in R4VaD and other relevant observational studies and Clinical Trials of Investigational Medicinal Products (CTIMPs) is encouraged as long as the two studies would not confound each other’s results, make attribution of adverse reactions difficult in the CTIMP or overburden participants. Specifically, to reduce burden on participants, data consistent with R4VaD and the co-enrolled study are shared; similarly results of tests performed clinically (e.g. MoCA) can be used in R4VaD and vice versa to avoid repetition and reduce workload for the patient and staff.

### Data capture and management

All data are entered into a secure password-protected electronic case record form (eCRF) hosted at the University of Nottingham. Baseline and 4–14-week follow-up data are entered by the local researchers at each participating hospital, whereas one and two-year follow-up data are entered by the central follow-up co-ordinators. All entries are validated, tracked and any changes are documented. Each participant’s data are anonymised, and participants are identified only by their Study ID number. Paper versions of the CRF are available to assist data collection which are filed in the patient’s folder and held securely at site. The eCRF includes range and validity checks, tracks missing/incomplete data and flags follow-up timepoints to aid the flow of data collection, study recruitment tracking and study management.

### Study assessments

Our approach to assessment recognises that different stages after stroke need specific approaches.^6^ We assess:

1. pre-morbid, i.e. peak adult cognitive ability;
2. pre-stroke cognitive decline;
3. post-stroke cognitive status at specific points after stroke to map cognitive trajectories;
4. demographic, clinical (current, past, pre-stroke medications and stroke-specific treatments), socioeconomic, lifestyle, neuropsychiatric symptoms including fatigue and functional status at relevant timepoints.

Our approach to assessment was informed by experts in stroke, cognition, neuropsychological assessment and lay advisors.^6,7^ We drew upon existing knowledge and best practice and implemented the following principles over multiple iterations and testings of the assessment schema: (a) avoid overburdening participants and carers, (b) avoid duplication, (c) each test is essential, (d) consistent across stages, (e) valid, with wide stroke usage^8^ for external comparison (e.g. we already have data on Telephone Interview for Cognitive Status (TICS) (cognition) and Zung (mood) for more than 7500 pts^9^) and (f) minimise known biases.^10,11^

We were guided by Cochrane Dementia, Dementia Platform UK (DPUK) Vascular Experimental Medicine Study Group^7^ and focus groups with study nurses and clinicians. People living with stroke have consistently recognised post-stroke cognitive problems as a priority.^5^ Our approach to R4VaD was also informed by our extensive work with stroke survivors and their caregivers. The R4VaD Participant Panel and participant groups in study sites commented on timing and duration of cognitive assessments, avoiding repetitiveness, care of patients with cognitive difficulties, importance of including all stroke severities, suitability of questions deemed to be quite personal, carer involvement and careful wording of study information to convey the work’s importance while not exacerbating worry in those recently overwhelmed by acute stroke. Research practitioners at recruiting sites also identified and shared good practice ideas via regular site meetings and electronically (https://stroke.nottingham.ac.uk/r4vad/live/r4vad_login.php).

 Our data collection focuses on efficiency (minimising test time and duplication), validity (systematic reviews of test properties,^6,12^ relevance to VCI^8^) and feasibility (postal or telephone versions available^13^). Since cognitive and neuropsychological batteries may become overly burdensome and unsuitable in patients with stroke, we use a ‘stepped approach’ with brief assessments covering core domains for all participants and more detailed test versions as feasible.^14,15^ Patients with aphasia, hemiparesis or hemianopia are encouraged to complete as much of the tests as they are able to; reasons for inability to complete tests are recorded. The neuropsychological effects of vascular disease include delirium, fatigue, apathy and mood disorders^16^ in addition to cognition, quality of life and well-being, providing comprehensive physical, functional, cognitive and neuropsychiatric data.

Our follow-up is flexible and includes face-to-face (although ideal, it is unfeasible in all, or in a study of this size), telephone or postal follow-up; online/email follow-up could be used. Combining phone and postal questionnaires allows a greater range of cognitive assessment than either alone (e.g. postal allows visuospatial tests), improves rates of data completion and reduces losses to follow-up. Failure to complete a test may reflect patient status and is thus an outcome in its own right, so we record non-testability, employing ‘intention to diagnose’ approaches to deal with test non-completion.^14^ Finally, informants know the patient well, can recognise change^12^ and are invaluable if communication problems preclude even brief direct-to-patient assessments. Therefore, we provide validated informant versions of questionnaires. Engagement with relatives and partners also increases retention and data completeness and provides data on care-giver strain.

**Figure 1.**
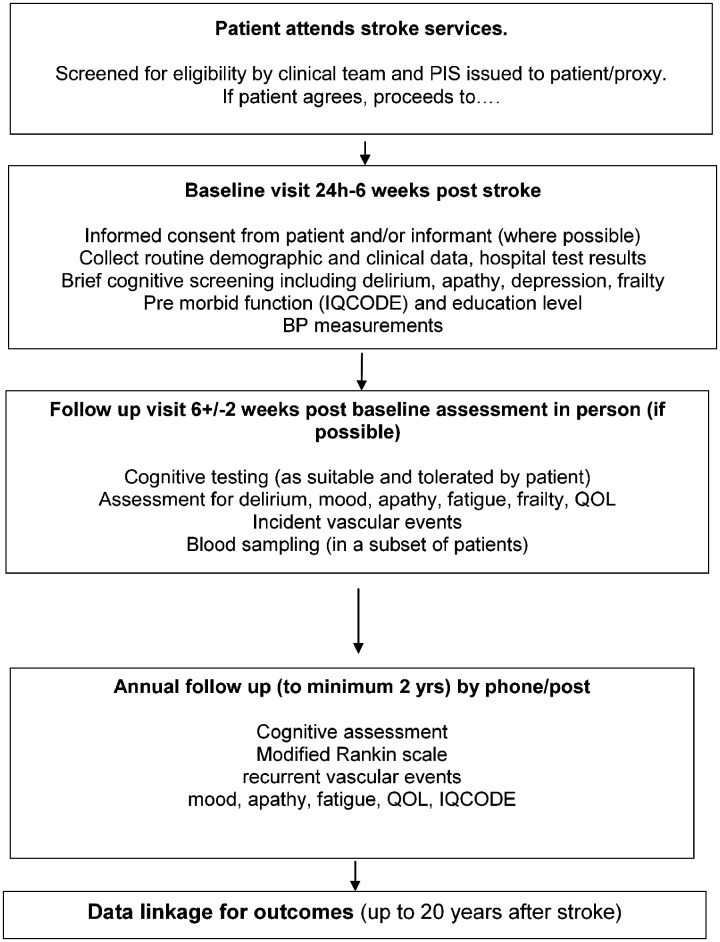
Flow chart of recruitment and study protocol.

Figure 1 summarises the study visits. Supplementary Table 1 shows the assessments performed at each visit, and Table 2 shows the validated tests used to perform these assessments.

**Table 2.** List of validated assessments.

Feature assessed	Main test	Abbreviation	Additional contributing tests
Functional outcome	Modified Rankin Scale^17^	mRS	SIS;^18^ Barthel; Lawton^19^
Quality of life	EuroQol-5D	EQ-5D	ONS-4
Wellbeing	Office for National Statistics Personal Well-being	ONS-4	
Basic and extended activities of daily living	Barthel^20^		Lawton^19^
Frailty	Clinical Frailty Scale^21^		
Cognition: multidomain screening	Montreal Cognitive Assessment,^22^ modified Telephone Interview for Cognitive Status^23^	MoCATICS-m	
Cognition: domain specific tests	Trail Making Test A and B, with timing and error counts;^24^ Letter Digit Coding;^25^ Boston Naming Test;^26^ verbal fluency for F, S and A;^27^ animal naming^26^	Trails A, BLDCBoston NamingAnimal naming	
Cognition: Informant-provided	Informant Questionnaire on Cognitive Decline in the Elderly^28^	IQCODE	
Anxiety and depression	Zung;^29^ Generalised Anxiety Disorder;^30^ Patient Health Questionnaire (PHQ)^31^	ZungGADPHQ	EQ-5D
Fatigue	Brief Fatigue Inventory	BFI	PHQ Zung^29^
Apathy	PHQ; Informant Neuropsychiatric Inventory Questionnaire Version^32^	NPI-Q	Zung^29^
Mobility, dexterity	Stroke Impact Scale^18^BarthelLawton	SIS	EQ-5D
Current Social Support	Social Support Scale^21^	SSS	
Delirium	4 A’s test^33^	4AT	
Educational exposure	Years in full time education; age leaving full time education		
Premorbid cognitive ability	National Adult Reading Test^34^	NART	Educational exposure
Current SES	Current occupation; postcode		
Childhood SES	Father’s and mother’s occupations		

SES: socioeconomic status.

We assess functional status, quality of life, well-being, basic and extended activities of daily living, mobility, frailty, cognition using multidomain screening tools and domain-specific tests, anxiety, depression, fatigue and apathy. Several questionnaires (Table 2) contribute several types of information. We record current social support, place of residence, educational exposure (to estimate premorbid cognitive ability), current and childhood SES. We use Informant-provided Informant Questionnaire on Cognitive Decline in the Elderly (IQCODE)^28^ to estimate pre-stroke cognitive decline and 4 A’s test (4AT) for delirium around the time of stroke.

*Baseline assessment* occurs as soon as possible, between 24 h to six weeks after stroke, the latter to enable patients to be included who were very ill in the first week but then start to recover. We record details of the index stroke including severity (worst and at the time of assessment with National Institutes of Health Stroke Scale, NIHSS) and evidence of delirium (clinical, from informant using 4AT), past medical and family history, lifestyle (smoking, alcohol and dietary salt intake and exercise), pre-stroke medication, treatment for the index stroke, visual and auditory impairments. Informants are asked about pre-stroke cognition (IQCODE^12^). BP is assessed using standard protocols and validated, calibrated monitors to obtain three measures at least one minute apart.^35^ Routine brain imaging (CT in most, MRI in some) is collected centrally to classify the index stroke (location, size, subtype, arterial territory) and other (prior stroke lesions, white-matter lesions, lacunes, atrophy in all; perivascular spaces, microbleeds, siderosis where MRI is available) findings using well-validated standard visual assessment tools.^36,37^ Bloods are taken for genetic analysis.

*Early follow-up* is at 6 ± 2 weeks post-baseline assessment (i.e. 4–14 weeks after stroke) by local site staff. At this stage, participants are more likely to be able to complete multidomain cognitive tests (Table 3)^8^ or shorter tests.^38^ The assessment coincides with local clinic review, those remaining in hospital being assessed in hospital. Contact by post or phone is also offered. We record place of residence, functional status or if the patient has died, the cause. Every effort is made to overcome barriers to follow-up (e.g. arranging transport if necessary; contacting the patient via the Informant, obtaining information from medical case-records and the Informant) and obtain reliable data. BP is assessed as at baseline. Bloods are taken for inflammatory markers (and genetics where not taken at baseline) and future analyses.

**Table 3.** Cognition categories and operational definitions for R4VaD primary endpoint.

Primary category	Operationalisation	Sub-category	Operationalisation
Normal cognition	No evidence of cognitive impairment (T-MoCA: 20–22 OR TICSm: 25–39)	Normal	
Minor Neurocognitive disorder (mild cognitive impairment)	Evidence of cognitive impairment (T-MoCA: 15–19 OR TICSm: 17–24) ANDNo evidence of functional impairment (mRS < 2 OR no change in mRS if pre-stroke mRS > 1)	Single domain	Scores are reduced by >1 point in only one cognitive domain of T-MoCA or TICSm
		Multi domain	Scores are reduced by >1 point in more than one cognitive domain of T-MoCA or TICSm
Dementia	Clinical diagnosis made independent of studyAny clinical diagnosis of dementia made by memory clinic (or equivalent, this would include primary care) Any recording of dementia on death certificationAny prescription of cholinesterase inhibitor or memantineORPre-stroke dementia (Baseline assessment IQCODE > 3.6 AND MoCA < 23) ORIn-study evidence of persisting multi-domain cognitive impairment (T-MoCA score <19 OR TICSm<24 on more than one annual follow-up) andEvidence of functional impairment (mRS ≥ 2 or IQCODE > 3.6 at final follow-up)	Mild	Cognitive impairments (T-MoCA 15–19 OR TICSm 17–23) ANDMinimal functional problems (mRS < 3)
		Moderate	More severe cognitive impairments^a^ (T-MoCA 10–14 OR TICSm 12–16) ANDMore limiting function (mRS 3 or 4) AND [Barthel > 60 (if available)]
		Severe	Severest cognitive impairments (T-MoCA < 10 OR TICSm < 12) AND Most limited functionCare-home admissionOR (mRS 4,5 OR Barthel < 60) ORAny NPI Q item score of 3
Death		Death	

Higher scores on cognitive tests indicate better cognitive function. ‘Equivalent’ will include any formal diagnosis of dementia or a dementia subtype made by a suitably trained professional; in the UK, this is likely to be a geriatrician, neurologist, old age psychiatrist or psychologist. At follow-up, the functional status (mRS and Barthel ADL) can be taken from either the participant or the informant, the more severe score should be used to inform the cognitive categorisation. MoCA can be used in place of T-MoCA where available.

^a^Possible scenarios that do not fit will be referred for expert panel consensus: e.g. (a) mild cognitive impairment, e.g. MoCA 15 but more severe disability mRS 3 or above which would imply that the disability is driven by physical rather than cognitive problems; (b) more severe cognitive problems but less severe disability e.g. MoCA 14 but mRS 2, which may in many cases represent under scoring of mRS since it seems implausible that a person could have no limitations in extended ADL with this level of cognitive impairment.

*Annual follow-up* (to two years minimum, maximum four years) is performed centrally by post and phone. We avoided online/email follow-up since only 53% of 1-adult and 85% of 2-adult households aged > 65 have internet (Office of National Statistics 2016).^39^ Central follow-up by validated telephone and post methods occurs at three centres, UCL, Leicester and Glasgow, spreading the study activities across core sites.

## Outcome measures

### Primary outcome

The primary outcome of R4VaD is cognitive decline or (incident and prevalent) dementia/major neurocognitive disorder up to at least two-years post-stroke assessed with a seven-point ordinal scale that includes death (Table 3). The ordinal scale maps onto the four-point DSM-5 criteria for major neuro-cognitive disorder (dementia) diagnosis but provides more granularity in the ‘dementia (major neurocognitive disorder)’ category to reflect practical implications. The seven-point ordinal scale incorporates cognition (using MoCA and TICS-m, so as to apply to all patients) and function (mRS, and Barthel), clinical diagnosis of dementia made outside R4VaD, place of residence and IQCODE. It also incorporates the number of affected domains of cognition (which may have practical implications e.g. if some domains are more restrictive on independence than others) and impact of the cognitive impairments on functional dependency.

This ordinal approach is pragmatic and uses all available data^40^ (including prescriptions) to categorise neurocognitive status while minimising the impact of missing or untestable data. Ordinal data facilitate sophisticated quantitative analyses (a comprehensive statistical analysis plan will be described in a separate paper). Finally, since this approach could be useful for future trials in VCI, R4VaD will assess ordinal classification and analysis approaches at scale.

*Secondary outcomes* include the following (the list is not exhaustive)

1. Death
2. Disability (mRS)
3. Function in activities of daily living (Barthel; Lawton; SIS)
4. Recurrent stroke or other vascular events
5. Domains of cognition (complex attention, executive, language, memory and visuospatial function), allowing for analysis at the individual domain level or at participant level creating normalised, aggregate ‘z scores’
6. Other neuropsychological outcomes: mood, majoring on depression and anxiety (Zung; PHQ; GAD), apathy (PHQ) and fatigue (PHQ; BFI)
7. Frailty (Clinical Frailty Score)
8. Quality of life assessment (EuroQol 5D) and well-being (Office for National Statistics Personal Well-Being)
9. Vascular measures: blood pressure (BP), carotid stenosis, vascular stiffness measures
10. Imaging findings (lesion location, size, pre-stroke changes), DTI, brain and lesion volumes
11. Inflammation (blood markers)
12. Genetics

### Sub-studies

An aim of R4VaD is to maximise value through addressing additional research questions. Thus, in addition to efforts to maximise use of the core data, consent for future studies, etc., several sub-studies were included in the original funding application, with the potential to add others. Those in the funding application include:

#### Inflammation

Blood-derived samples are stored for current and future discovery analyses at Manchester University. Planned analyses focus on a high sensitivity assay of the inflammatory cytokine IL-, which is thought to be involved in cognitive decline including PSCI^41^ using single-molecule counting technology (Singulex) to detect extremely low cytokine levels; blood is stored for analysis of other cytokines and proteins.

#### Genetics

Genetic susceptibility is important in Alzheimer’s disease (AD) and stroke and likely to be important but under-studied in VaD. Blood is taken for DNA (from all possible participants) and transferred to Cambridge University for GWAS, with standard QC, imputation and statistical analysis methods to compare genetic profiles with and without PSCI. We will estimate genetic heritability for PSCI and identify SNPs significantly associated with dementia at the genome wide significance level of 5 × 10^−8^. Combining the anticipated 1000+ highly phenotyped patients from R4VaD with other Consortia will provide ∼5,000 patients with relevant post-stroke outcomes, making a crucial contribution to the global cohort.

#### Neuroimaging

In centres able to perform MR diffusion tensor imaging (DTI), up to 400 patients will have DTI and structural sequences (T1-, T2-, fluid-attenuated inversion recovery and susceptibility-weighted imaging) to assess several objective DTI tissue parameters (fractional anisotropy, mean, axial and radial diffusivity, peak width of skeletonised mean diffusivity^42^) to test sensitivity for predicting cognitive dysfunction compared with visible lesions (infarct size and/or location, WMH, lacunes, perivascular spaces, microbleeds, siderosis), brain volume loss or total SVD and frailty scores.^43^ The MRI data are collected centrally to analyse the DTI data and quantify visual features.^37^

### Efforts to minimise bias

#### Site training

All sites undergo standard set-up procedures to ensure appropriate capabilities, resources and recruitment numbers. UK Stroke Research Practitioners are trained in screening all patients assessed and diagnosed with stroke, determining the NIHSS and mRS, and are commonly experienced in EQ-5D and Barthel scales and cognitive testing. Sites receive training in R4VaD recruitment, administering the study questionnaires including cognitive and neuropsychiatric tests, completing the CRF, methods to encourage participant retention and ensure data completeness, in blood and imaging data acquisition, processing and transfer for central storage and analysis. A manual of cognitive testing, responses to frequently asked questions and training slides are provided to sites.

#### Consensus cognition diagnosis

Data from a subsample of those reaching one and two-year follow-up will be assessed by an expert multidisciplinary panel to assign a definitive diagnosis of cognitive status (Table 3) and likely aetiology.

#### Data linkage

We will link unanonymised data to ascertain recurrent stroke, dementia, death, place of residence and vital status and limit losses. We will use primary care and secondary care records and national registry data to ascertain long-term outcomes to supplement follow-up information to two years and thereafter.

We will assess external validity of the participants included in the cohort through comparisons with the Sentinel Stroke National Audit Program (SSNAP; England, Wales and Northern Ireland) and the Scottish Stroke Care Audit, and with screening logs at a subset of centres, with actions as necessary during recruitment to encourage a representative sample.

#### Sample size estimates

We used meta-analyses and trial data to determine sample size and provide scenarios of rates of PSCI across different patient characteristics (Table 4).

**Table 4.** Sample size estimations.

	Stroke severity		Total sample *N*
Outcome at one year after stroke	Mild	Severe	
Dementia (%)	10	20	572
Dementia (%)	10	15	1914
Dementia (%)	20	40	236
Dementia (%)	20	30	825
Dementia (%)	20	25	3008
Dementia (%)	20	27	1596

SSNAP provides data on numbers of patients admitted to hospital in England and Wales and showed that about 7000 stroke patients are admitted to the applicants’ hospitals/year with about 5700 alive at discharge,^44^ and an additional approximate 1000 (minor strokes) are seen as outpatients. The numbers of TIAs are not available for all sites. Thus, recruitment of over 1000 patients (20%; 125/centre) is feasible in one year and about 2000 in two years (250/centre), i.e. 1600–1700 ischaemic strokes and about 400 ICHs, with streamlined, light-touch approaches. This sample would recruit about 20% of patients admitted per applicant centre and is a conservative recruitment estimate for an observational study with very broad entry criteria. In addition, it would almost double data on PSCI after ICH.^45^

**Figure 2.**
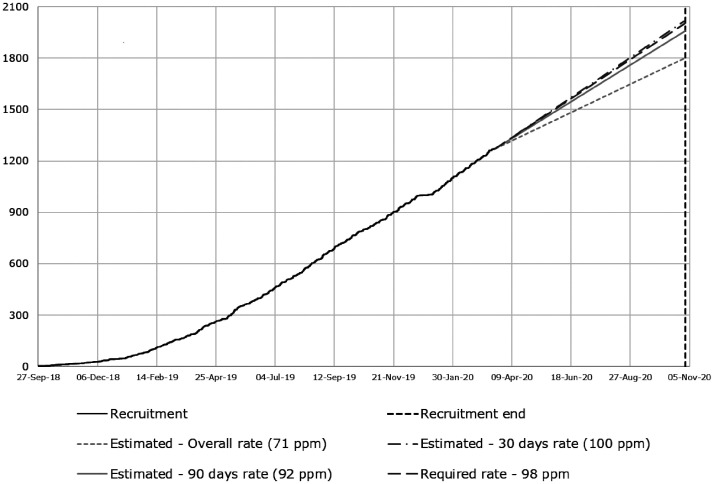
Recruitment actual versus target as at 20 March 2020.

Following considerable interest in the study from CRN-Stroke sites prior to the funding award, in addition to seven applicant sites, we expected to include up to 15 other NIHR Clinical Research Network Stroke Sites able to recruit in total about 1000 patients per year, thus potentially recruiting more than 2000 or completing recruitment of 2000 participants faster (Figure 2).

We estimated that, at power 0.90, *α* = 0.05, we could detect the following differences in dementia incidence at one year in mild versus severe stroke, respectively: 20% vs. 27%, *n* = 1596; 10% vs. 15%, *n* = 1914; 10% vs. 20%, *n* = 572 (Table 4). Thus, 2000 patients, with a wide range of stroke severities, will allow us to detect small (5% absolute), clinically meaningful differences in dementia between mild versus severe stroke, although the difference in dementia between mild and severe is likely to be larger (10+% difference).^46^ A sample of about 2000 will be able to detect differences in degrees of VCI and dementia and by subgroups such as age, pre-morbid cognitive ability, stroke subtype or vascular risk factors in multivariable models.

## Statistical analyses

The statistical analysis plan will be published separately.

### Study organisation and funding

R4VaD has a Study Steering Committee (SSC) including an independent chair, the applicants, funder representatives, an external expert and user representatives. The SSC meets six monthly to review study progress and are consulted ad hoc as necessary. The Study Management Group meets monthly by phone or in person. The Sub-studies Committee communicates ad hoc by email and phone to assess proposals.

In addition to regular email and phone communication between the Study Manager, other core study staff and research practitioners of the Clinical Research Network (England, Wales, Northern Ireland) and Stroke Research Network (Scotland), a teleconference is held every four to six weeks for all research practitioners at any sites to keep up to date with study procedures and share best practice. An R4VaD Newsletter is circulated regularly.

The Participant Panel communicates by email and teleconference and is consulted on an ad hoc basis for matters concerning study design, acceptability or to comment on potential research proposals arising from R4VaD.

An Investigator meeting is held annually in December.

The work is organised in Work-packages (Study Management including eCRF, Cognition, Inflammation, Genetics, Statistical analysis) located between steering committee member sites.

R4VaD is funded by a Priority Programme Award in VaD from The Stroke Association, British Heart Foundation and Alzheimer’s Society (Grant No. 16 VAD 07) and also supported by the Medical Research Council through the Dementia Platform UK.

### Data access

After study completion, data cleaning and lock, anonymised data will be available for secondary uses. Blood, genetic and imaging samples will be accessible for secondary uses. A Sub-studies sub-Committee of the R4VaD SSC considers proposals for sub-studies.

### Impact of SARS-CoV-2

The UK started lockdown in March 2020 due to the SARS-CoV-2 (COVID-19) pandemic, the Sponsor suspended recruitment on 18 March in line with Government rules and the NIHR Urgent Public Health Committee rule to only allow research addressing COVID-19 to continue. For participants who had been recruited shortly before the lockdown, we continued early follow-up as phone or post (which was already allowed), and all annual follow-ups were already by phone/post and continued uninterrupted. However, between January and March 2020, we had already been detecting impacts of the developing pandemic on participants who expressed increasing levels of anxiety and in suicidal thoughts and serious issues with interruptions to social support, particularly from those who had reached the one-year follow-up assessment at that time. Additionally, in view of the associations between cardiovascular risk and severe illness in those infected with SARS-CoV-2, we applied for regulatory approvals to continue the study to collect data on SARS-CoV-2 exposure, and symptoms, severity and treatment received if infected, at presentation with stroke and at annual follow-up in all participants. We also modified the data collection procedures accordingly. We submitted the applications to the four devolved UK administrations (Ethics and Research and Development Offices) and the NIHR Urgent Public Health Committee on 3 April 2020. Approval was granted in Scotland and England on 6 and 22 April, respectively, and the study restarted recruitment, during the pandemic lockdown, in Scotland on 17 April and in England on 6 May in the (so far) 11 sites where resource availability made it feasible to do so. Since lockdown was eased in the UK in early July 2020, all other sites are gradually opening.

## Discussion

R4VaD is a prospective observational, longitudinal inception cohort with central follow-up being carried out in major UK stroke centres representing geographic and socioeconomic diversity. Recruitment started on 25 September 2018 in Edinburgh; by 13 March 2020, 1271 participants had been recruited across 53 sites (Figure 2). The mean age is 69.8 years (SD13.3), recruited at median 7.0 (IQR 3.0–17.0) days post-stroke, most have ischaemic stroke (83%), 7% lack capacity; and 42% have an informant. Recruitment will continue for two years or until at least 2000 patients have been recruited. Note that on 18 March 2020, the study temporarily suspended recruitment due to Government restrictions from the SARS-CoV-2 pandemic, but re-opened to recruitment in April 2020, as detailed above, and has now recruited 1416 participants, i.e. 142 during the pandemic lockdown in the 11 of 53 centres that had resource available to continue. We will continue hereon collecting details on SARS-CoV-2 on all participants, including those recruited prior to the pandemic, enabling a before, during and after the pandemic analysis. While the pandemic has affected stroke services and research in all countries affected by SARS-CoV-2 and has undoubtedly delayed recruitment to R4VaD, we hope that the additional information gained by collecting data on SARS-CoV-2 across the UK will provide valuable additional information in the early and late impacts of the measures taken in the UK to address the pandemic and the infection itself on a vulnerable and high risk group of patients.

Fundamental gaps in knowledge on stroke, cognition and dementia were highlighted recently.^1,15^ Dementia varies from 7 (population studies, first stroke, no pre-stroke dementia) to 41% (hospital studies with recurrent stroke and pre-stroke dementia) at one year,^2^ but with confidence intervals spanning two to three-fold differences in dementia that are largely unexplained. Prevalence of MCI (29–68%) and dementia (8–22%) after TIA are variable and based on few patients.^47^ The aetiology, risk factors and prognosis of PSCI are poorly understood. They lack information on stroke subtype, e.g. lacunar stroke or ICH,^45^ progression of VCI to dementia^15^ or PSCI rates beyond the first year after stroke.^2,48^ Few studies consider pre-morbid intelligence^49^ or failing cognition pre-stroke,^3^ yet both factors affect risk of stroke and of PSCI. While the APOE-ε4 genotype is a major risk factor for AD with an allelic frequency of 15% in whites, in patients with TIA or stroke-only APOE-ε4, homozygosity was associated with increased risk of dementia post-stroke yet was present in less than 2% of patients.^50^ Cerebral amyloid angiopathy (CAA), a vascular disease which causes stroke and dementia, apparently is also not associated with the APOE-ε4 genotype, even in patients with severe CAA.^51^ R4VaD should progress understanding of different contributors to cognitive impairment in the presence of cerebrovascular disease.

The limited data on the complex interplay between individual risk factors and PSCI or dementia, how risk factors affect dementia pathophysiology, or brain health and resilience,^52^ make it difficult to advise individuals, plan randomised clinical trials or develop clinical services. Generalisability of data is restricted by selection bias, suboptimal testing and attrition.^4^ Cognitive testing is recommended in UK stroke guidelines,^53^ but many tests are impractical for stroke or insensitive to VCI.^4,15^ Lack of proven clinical utility may explain why many GPs do not perform cognitive screens routinely. Use of different cognitive tests inflates variance in VCI/dementia rates, hampers between-study comparisons and efforts to understand mechanisms, as individual studies lacked vascular risk factor adjustment; similarly, systematic reviews^2^ and routine health data^54^ disagree on the importance of common risk factors. Cognitive testing alone does not capture the psychological sequelae of stroke: fatigue, apathy, mood^16^ affect cognition; in turn, three-month cognition scores correlate highly with dependency, mood and quality of life.^46^

A broad stroke cohort in R4VaD will capture all potential VaD phenotypes (small vessel disease, multiinfarct, strategic infarct, mixed pathologies),^4^ strengthening knowledge on VaD. We will collect cognitive information across a continuum of stroke severities because cognition is relevant after all severities of stroke.^2^ The prospects for physical recovery, even after severe stroke, are changing radically with thrombolysis, thrombectomy, improved discharge support and community rehabilitation, but with unknown impact on cognition. Improved physical recovery from initially severe stroke may unmask potentially limiting cognitive deficits and equally, deficits in mild stroke may be missed by conventional clinical services yet restrict independence.^48^

R4VaD, designed to address these highlighted issues, will assess long-term cognition after stroke in a broad, inclusive population, along with providing valuable information on mechanisms and risk factors. The achievement of recruitment targets and completeness of baseline and follow-up data will give practical evidence of the acceptability, feasibility and practicality of the assessments ‘in the field’. We acknowledge the mortality and attrition associated with trying to include all severities of stroke, so have included multimodal patient-focussed follow-up to minimise losses, and analysis methods to account for competing risk biases associated with early mortality.

R4VaD will provide well-phenotyped patients (including cortical and subcortical VaD/VCI), stratified and consented for re-contact for future trials, which is particularly important given the recent difficulties in recruiting patients with VCI into trials. Furthermore, patients with stroke represent an ‘enriched’ sample, similar to the relationship between MCI and AD. R4VaD embeds priority sub-studies on inflammation and genetics, and we store blood samples for future discovery. R4VaD will help validate the proposed staged approach to cognition and related assessments, for research and clinical use. R4VaD will provide data to inform service design and assist those at risk of PSCI to plan their future.^5^ R4VaD combines the UK’s considerable stroke research strengths, which have helped transform stroke care into co-ordinated prevention, treatment and recovery in the last 25 years, with the dementia research expertise of the DP-UK and Dementia Research Network.^52^ Although stroke and dementia services and Research Networks currently operate in completely different ways (different hospitals, time frames, scheduling, patient caseload), we hope that R4VaD will encourage clinical services and research sites for dementia and stroke to operate more closely together since the two disorders’ risk factor profiles and impacts on daily life are so closely intertwined.

## Supplemental Material

sj-pdf-1-eso-10.1177_2396987320953312 - Supplemental material for Rates, risks and routes to reduce vascular dementia (R4vad), a UK-wide multicentre prospective observational cohort study of cognition after stroke: Protocol Click here for additional data file. Supplemental material, sj-pdf-1-eso-10.1177_2396987320953312 for Rates, risks and routes to reduce vascular dementia (R4vad), a UK-wide multicentre prospective observational cohort study of cognition after stroke: Protocol by Joanna M Wardlaw, Fergus Doubal, Rosalind Brown, Ellen Backhouse, Lisa Woodhouse, Philip Bath, Terence J Quinn, Thompson Robinson, Hugh S Markus, Richard McManus, John T O’Brien, David J Werring, Nikola Sprigg, Adrian Parry-Jones, Rhian M Touyz, Steven Williams, Yee-Haur Mah, Hedley Emsley and the R4VaD Investigators in European Stroke Journal
