# Supplementary material for: Rates, risks and routes to reduce vascular dementia (R4vad), a UK-wide multicentre prospective observational cohort study of cognition after stroke: Protocol
Source: Eur Stroke J. 2020 Oct 11;6(1):89–101. doi: 10.1177/2396987320953312 (PMC7995325; doi:10.1177/2396987320953312)
Supplement: sj-pdf-1-eso-10.1177_2396987320953312 - Supplemental material for Rates, risks and routes to reduce vascular dementia (R4vad), a UK-wide multicentre prospective observational cohort study of cognition after stroke: Protocol [file sj-pdf-1-eso-10.1177_2396987320953312.pdf]

Appendix: The R4VaD Investigator Group

Writing panel: JM Wardlaw, F Doubal, R Brown, E Backhouse, L Woodhouse, P Bath, T Quinn, T Robinson, HS Markus, R McManus, DJ Werring, JT O'Brien, N Sprigg, A Parry-Jones, R Touyz, S Williams, YH Mah, H Emslie

Study Steering Committee: G Ford (Independent Chair), Edo Richard (Independent Advisor), JM Wardlaw, F Doubal, R Brown, E Backhouse, L Woodhouse, P Bath, T Quinn, T Robinson, H Markus, R McManus, DJ Werring, JT O'Brien, N Sprigg, A Parry-Jones, R Touyz, S Williams, YH Mah, H Emslie.

Statistician: L Woodhouse (Nottingham).

Study Manager: Rosalind Brown, Ellen Backhouse (Edinburgh).

Programmers: eCRF, Richard Dooley, Lee Haywood (Nottingham); image data housekeeping system, David Buchanan, David Perry, and image review system, Jeb Palmer (Edinburgh),

Image data manager: Eleni Sakka (Edinburgh)

Study Co-ordinators: Rachel Penman (Edinburgh), Ruth Graham (Glasgow), Matthew Mills (Salford), Lucy Fleming (Nottingham), Jennifer Mitchell-Douglas (Cambridge), Maria Pardo Garcia (KCL), Kyriaki KaraliTsilimpari (Leicester), Hatice CApar (UCL)

Follow-up Co-ordinators: Hatice Capar (UCL, London), Ahtasam Ebraimo (Leicester), Louise Craig (Glasgow)

Participant Panel: [to be added once confirmed]

Site Investigators, Principal Investigators (PIs), Research Associates (RAs) and Research Nurses (RNs):

Applicant sites, England:

Nottingham: PI Ashit Shetty; RN Lucy Fleming

Preston: PI Hedley Emsley; RN Bindu Gregary, Anne-Marie Timoroksa

Salford: PI Adrian Parry-Jones; RNs Matthew Mills, Bethan Charles, Stephanie Lee

KCH (PRUH & Denmark Hill), London: PI Yee Mah; RNs: Maria Pardo Garcia, Myriam Aissa, Con Tibajia, Staci Conway

Leicester: PI Amit Mistri; RNs Kyriaki KaraliTsilimpari, Shagufta Khan

Cambridge: PI Jennifer Mitchell-Douglas; RN Elaine Amis

UCL, London: PI David Werring; RNs Hatice Ozkan, Sabaa Obarey, Francia Nina, Florence Mantey, Marilena Marinescu

Applicant sites, Scotland:

Edinburgh: PI Fergus N Doubal; RA, Ellen Backhouse; RNs Rachel Penman, Allan Macraild,

Glasgow: PI Terry Quinn; RNs Ruth Graham, Louise Craig

Sites in NIHR Clinical Research Network, England:

Birmingham: PI Sissi Ispoglou; RN Anne Hayes, Steven Hurdowar

Royal Devon and Exeter: PI Jane Sword; RN Angela Bowring, Alina Glover

Bury: PI Narayanamoorthi Saravanan; RN Pamela Bradley, Clair Carty

Poole: PI Suzanne Ragab; RN Judith Dube

Yeoville: PI Khalid Rashed; RNs Natalie Guilemer , Alfonso Tanate, Sarah Board, Christine Hellyer, Carinna Vickers

St Georges: PI Liquin Zhang; RNs Cai Sim, Lauren Binnie, Rebecca Williams

Leeds: PI Ahamad Hassan; RN Dean Waugh

Sheffield: PI Kirsty Harkness; RN Jo Howe, Christine Kamara

St Thomas': PI Hannah Millitor; RN Meegan Gibbons, Hannah Molitor

Kings Lynn: PI Umesh Rai; RN Tracy Fuller

Southampton: PI Richard Marigold; RN Simon Smith, Jake Harvey, Laura Baldwin

Kingston: PI Asis Kumar; RN Kathryn Sollesta

Chester: PI Arumugam Nallasivan; RN Michaela Martin

Telford: PI Meena Srinivasan; RN Nicki Motherwell

Wirral: PI Ruth Davies; RN Venetia Johnson, Rachael Fergusson

Rotherham: PI Dawn Collier; RN no recruitment

Blackburn: PI Sheeba Philip; RN Stephen Duberley

Bath: PI Andrew Stone; RN Lauren Pearce, Thelma Costa

Pilgrim Hospital: PI David Mangion; RN no recruitment

Lincoln: PI Kresimir Jergovic; RN Sarwat Arif, Susie Butler

West Suffolk: PI Abul Azim; RN Lisa Wood

Durham: PI David Bruce; RN Ami Wilkinson, Gill Rogers

Hull: PI Rayessa Rayessa; RN Emma Clarkson

Broomfield Hospital: PI Ramanathan Kirthivasan; RN Caroline Fox, Victoria Mead

St Peter's Hospital: PI Giosue Gulli; RN Emma Young, Margaret D'Souza

Scarborough: PI El Nour; RN Lauren Barman

Lancaster: PI Pradeep Kumar; RN Charlotte Culmsee, Rebecca Anderson

Stepping Hill: PI Joseph Vassallo; RN Clare Tibke, Liane Marsh

Coventry: PI Bander Dallol; RN Alison Hanson, Michelle Clarke, Kimberley Yates

Gloucestershire Royal Hospital: PI Dipankar Dutta; RN Deborah Ward

Royal Victoria Hospital: PI Anand Dixit; RN Valerie Hogg, Julie Parker

Bradford Royal infirmary: PI Chris Patterson; RN Outi Quinn, Ruth Bellfield

Pinderfields Hospital: PI Prabal Datta; RN Ann Needle, Shannen Beadle

Charing Cross Hospital: PI Joseph Kwan; RN Peter Wilding

Sites in Clinical Research Network, Scotland

Aberdeen: PI Joan Macleod; RN Vicky Taylor

Dundee: PI Sarah Ross; RN Claire Whyte, Louise Cabrelli

Kircaldy: PI Vera Cvoro; RNs Amanda McGregor, Mandy Couser

Monklands: PI Mark Barber; RN Derek Esson

Perth: PI Sarah Ross; RN Mairi Stirling

QEUH, Glasgow: PI Jesse Dawson; RN Elizabeth Colquhoun

Sites in Clinical Research Network, Wales

Morriston Hospital: PI Peter Slade; RNs unnamed

Hengoed Ysbyty Ystrad Fawr: PI Jonathan Hewitt, RN Gail Marshall, Rebecca Wallace, Sean Cutler

Wrexham: PI Walee Sayed; RN Rachel Hughes
